# The Use of Deep Learning-Based Intelligent Music Signal Identification and Generation Technology in National Music Teaching

**DOI:** 10.3389/fpsyg.2022.762402

**Published:** 2022-06-22

**Authors:** Hui Tang, Yiyao Zhang, Qiuying Zhang

**Affiliations:** ^1^School of Arts, Hunan City University, Yiyang, China; ^2^Department of Music, Chugye University for the Arts, Seoul, South Korea; ^3^College of Art and Communication, Beijing Normal University, Beijing, China; ^4^College of Art, Yunnan Minzu University, Kunming, China

**Keywords:** deep learning, music style, Long Short-Term Memory network, psychology, quality education

## Abstract

The research expects to explore the application of intelligent music recognition technology in music teaching. Based on the Long Short-Term Memory network knowledge, an algorithm model which can distinguish various music signals and generate various genres of music is designed and implemented. First, by analyzing the application of machine learning and deep learning in the field of music, the algorithm model is designed to realize the function of intelligent music generation, which provides a theoretical basis for relevant research. Then, by selecting massive music data, the music style discrimination and generation model is tested. The experimental results show that when the number of hidden layers of the designed model is 4 and the number of neurons in each layer is 1,024, 512, 256, and 128, the training result difference of the model is the smallest. The classification accuracy of jazz, classical, rock, country, and disco music types can be more than 60% using the designed algorithm model. Among them, the classification effect of jazz schools is the best, which is 77.5%. Moreover, compared with the traditional algorithm, the frequency distribution of the music score generated by the designed algorithm is almost consistent with the spectrum of the original music. Therefore, the methods and models proposed can distinguish music signals and generate different music, and the discrimination accuracy of different music signals is higher, which is superior to the traditional restricted Boltzmann machine method.

## Introduction

In the Internet age, the concept of “music without borders” is accepted by more people. There are some differences in music expression in different countries and regions, and the thoughts and emotions contained in music can always resonate with people. Music fully expresses its value in human life (Yuan and Wu, 2020). Music production is a way of artistic expression of people’s thoughts and feelings with music as the carrier. Therefore, the music contains people’s most sincere feelings, which need people to feel through hearing. Research on the psychological changes in music teaching is conducive to understanding the changes in individual behavior and psychological cognition in the process of teaching.

There are countless kinds and quantities of musical instruments in the world, and the storage methods of music files have also become diversified. Music genres have gradually formed based on the emergence of musical instruments and the diversification of music storage methods. Jazz, classical music, pop music, hip-hop, and rock music have become familiar words. Now, the traditional music arrangement and music information retrieval have been gradually replaced by computer technology. Digital audio processing, speech recognition, speech compression coding, and text speech conversion have become increasingly diversified and accurate under the revolution of information technology. Sitaula et al. (2021) studied the application of neural network technology to the classification of intestinal peristaltic and non-peristaltic sounds. They optimized the classification results by Laplace hidden semi-Markov model. The experimental results show that this method can enhance the accuracy of bowel sound detection and promote the possibility of telemedicine application in neonatal nursing in the future. With the wide application of machine learning (Ma et al., 2021) and deep learning (DL) (Quazi et al., 2021) in face recognition, speech recognition, and image recognition, people are gradually trying to apply this technology to the field of music generation. Hongdan et al. (2022) studied the application of machine learning technology to the recognition and classification of recording genres. Moreover, a model based on a convolutional neural network (CNN) was proposed to identify the spectrum of recorded audio through training. Moreover, the time and frequency domains’ features were extracted from the audio signal, combined into the machine learning model, and trained to classify the audio files. The research shows that classification accuracy is largely affected by feature selection for classification. In some classification systems, training errors may affect the model’s output. Nag et al. (2022) proposed an algorithm model based on CNN to identify the emotion of the music contained in Indian classical music. The database of 1,600 emotion fragments extracted from Indian classical music was established, and the emotion in music was classified by the method based on CNN. Li et al. (2022) studied the application of deep neural networks to music classification and used a spectrum diagram to evaluate the model’s performance. The music audio file was converted into a spectrum through modal transformation, and then the music was classified through DL. The experimental results show that the experimental results of the proposed model are always better than those of other neural network models. Deep learning is more powerful than machine learning in storing and processing massive data (Wu W. et al., 2020). Hence, more deep neural networks are used in music analysis and processing, especially Recurrent Neural Network (RNN) and Long Short-Term Memory (LSTM) networks.

RNN is first applied to music classification, but the effect is not very ideal. Due to the large correlation between the front and back notes, the data of the previous time or earlier cannot be obtained by using ordinary RNN, which makes the classification effect or the acquisition of musical features such as tone, timbre, loudness, and rhythm inaccurate. People have improved RNN and added forget gate on the original basis. It overcomes the problem of recording the connection between long spatiotemporal data, enables RNN to record previous relevant data information, and successfully overcomes the problem of long-time and spatial sequence. At present, more people use the LSTM network for emotion analysis and processing and some intelligent recommendation models. Neural network technology is used to achieve intelligent music recognition, and the ability of the designed algorithm to deal with related problems is improved by optimizing the recognition process. DL technology will be adopted to realize the intelligent recognition and generation of music signals and improve the algorithm’s output by optimizing the model parameters and structure.

## Materials and Methods

### Analysis of National Music Teaching Based on Psychology

Music education psychology is the research on the changes in psychological activities in music teaching. It is the product of the combination of psychology and education. Besides, psychology can be adopted to study the changes in people’s psychological law in teaching. In cognitive psychology, the occurrence and defense of feelings such as feeling, attention, consciousness, knowledge, and gene can be systematically explained to provide a reference for people’s research on cognitive activities such as imagination, meaning, and thinking. The psychological activities related to cognitive psychology and sound provide a basis for studying new three-dimensional characters in national music teaching.

Music content and emotional expression are crucial contents running through the three links of creation, performance, and listening. Each link gives music special significance and vitality, and involves cognitive activities in people’s feelings, perceptions, and consciousness. Wu et al. (2018) studied the application of information and communication technology to traditional teaching to improve the effectiveness of teaching and training (Wu et al., 2018). Hence, the effect of introducing information technology and DL into the national music teaching classroom is analyzed by combining educational psychology to effectively make an accurate judgment on the application of intelligent music signal identification and generation technology in national music teaching (Wu and Song, 2019). Hence, psychology can be employed to analyze the psychological cognitive process of applying DL technology to national music teaching.

### The Music Genre and Timbre Characteristics

The most basic part of identifying music signals is the classification of music genres. Music is divided into multiple genres according to its characteristics, and the characteristics of different genres are also quite different (Jiang et al., 2020). However, music genres have similarities and differences (Kim and Oh, 2021). At present, the most widely recognized classification structure in the world mainly includes the GTZAN Genre and ISMIR2004 Genre. The GTZAN Genre mainly divides music genres into 10 categories: blues, country, hip-hop, jazz, pop, disco, classical, rock, reggae, and metal. The ISMIR2004 Genre mainly divides music into six genres: classical, electronic, jazz/blues, metal/punk, and rock/pop (Ng et al., 2020).

Music characteristic is the embodiment of the essential attribute of music. It is crucial to extract the characteristics of music to distinguish different music styles and genres (Caparrini et al., 2020). At present, there are two main types of music features: physical features and time-domain features. Short-term features are divided based on human sensory characteristics, mainly including tone, timbre, and loudness, which can be expressed by specific numerical features (Wick and Puppe, 2021). Some time-domain features cannot be expressed by specific numbers. The details are as follows:

(1) The short-time energy represents the amplitude of the music signal at a certain time. The calculation reads:


(1) $$\begin{array}{c} \omega_{n}=\sum_{m=-\infty }^{\infty }{\left[\theta \,\left(m\right)\,\phi \,\left(n-m\right)\right]}^{2}\\ =\sum_{m=n-\left(N-1\right)}^{n}{\left[\theta \,\left(m\right)\,\phi \,\left(n-m\right)\right]}^{2} \end{array}$$

*n* represents the *n*-th sampling point, θ(*m*) is the signal value of the sampling point, ϕ(*n*−*m*) represents the window function, and *A* donates the window length (Wu and Wu, 2017).

(2) A crucial index to measure the high-frequency component of a signal is the short-time average cross zero ratios. In the waveform analysis, the more the high-frequency components are, the more the zero-crossing times are. Equation (2) donates this feature:


(2) $$\lambda_{n}=\frac{1}{2\,A}\,\sum_{m=n-\left(N-1\right)}^{n}\text{sgn}\,\left[\theta \,\left(m\right)\right]-\text{sgn}\,\left[\theta \,\left(m-1\right)\,\phi \,\left(n-m\right)\right]$$

λ_*n*_ represents the short-time zero-crossing rate. θ(*m*) donates the signal value of the sampling point. ϕ(*n*−*m*) is the window function, and *A* is the window length; sgn represents the symbol function, that is, the value is 1 when *x*(*m*)≥0. Otherwise, the value is 0.

### Musical Instrument Digital Interface Music

Audio storage formats mainly include MP3, Windows Media Audio (WMA), MIDI, and WaveForm (WAV). There are usually three types of events in music processing, MIDI events, system-specific events and meta events (Wu et al., 2019). The storage of audio is of great significance for music discrimination and generation (Siphocly et al., 2021). MIDI music is adopted here.

### Design of Music Style Recognition and Generation Model

**FIGURE 1:**
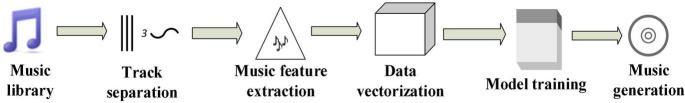
Flow chart of music style recognition and generation model.

The most crucial thing in music signal identification is the identification of music style (Ramírez and Flores, 2020). The function of the model is to extract music features from different styles and genres of music in the music library through track separation technology, perform vectorization processing of the extracted music feature data, and then train the model. The LSTM network in DL is mainly adopted to enable computers to produce different styles of music (Sun et al., 2020). Figure 1 displays a flow chart.

### Data Preprocessing

**FIGURE 2:**
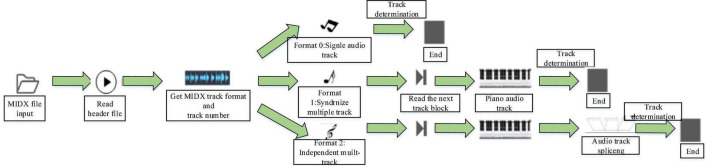
Flow chart of data preprocessing.

Music data can be trained after processing (Hughes et al., 2018). In this model, track separation, music feature extraction, and data vectorization are mainly performed on the data (Gunawan et al., 2020). Figure 2 presents the architectural design of the model. MIDI music contains three data types, represented by 0, 1, and 2; 0 means that there is only one track; 1 means that multiple tracks will start playing in the same time series and at the same beat; 2 means that multiple tracks can be selected freely without starting simultaneously. Singletrack and independent multi-track MIDI files are relatively simple to extract. Since the tracks of independent multi-tracks are independent, the header files of the tracks need to be traversed in turn (Chen, 2019). All tracks of the music in format 1 are synchronized, so it is troublesome to separate, and the tracks need to be spliced. Music features are usually divided into physical features and time-domain features. Time-domain features can be only displayed by specific instruments (Rogoza et al., 2018). To better obtain the effective loudness, MIDI music extraction data containing at least 45 different loudness (the number of each loudness exceeds 20) are selected to become effective music data (Wu Y. J. et al., 2020). The effective music data are converted into a music score matrix as the input vector of the network. Meanwhile, the music score matrix needs to reflect the structure of MIDI and the characteristics of music in the form of vectors.

### Design of Music Genre Generation Model

**FIGURE 3:**
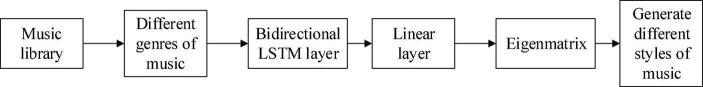
Flow chart of music genre generation model.

The music library contains music of various genres, and each genre has the same style. The system mainly needs to acquire the music characteristics of MIDI music in different genres, train the network, and then generate music of different styles (Zheng et al., 2018). The algorithm includes a music genre analysis model and a music style generation model. Figure 3 presents the model architecture. The music genre analysis model divides the learning problem into two parts. The first part is employed to learn the music features in the score and convert them into feature vectors, and the second part is to obtain the range of music intensity (Ahn et al., 2020).

Different genres of music have different musical characteristics. In this system, MIDI music of piano is mainly used. The main function of the music genre analysis model is to learn the style of music by learning a specific music genre, such as jazz, pop, and classical. (Yang and Lerch, 2020). Finally, different music genres will be distinguished according to the different music intensities. The music intensity matrix can be generated in the prediction part. The model mainly includes a bidirectional LSTM network layer and a linear layer (Hawley et al., 2020). The input of the model is a specific genre of music. Feature learning is carried out through a bidirectional LSTM network. The matrix containing sound intensity is generated through the linear layer. Finally, it is converted into music with the musical style that can be played (Chang et al., 2021).

**FIGURE 4:**
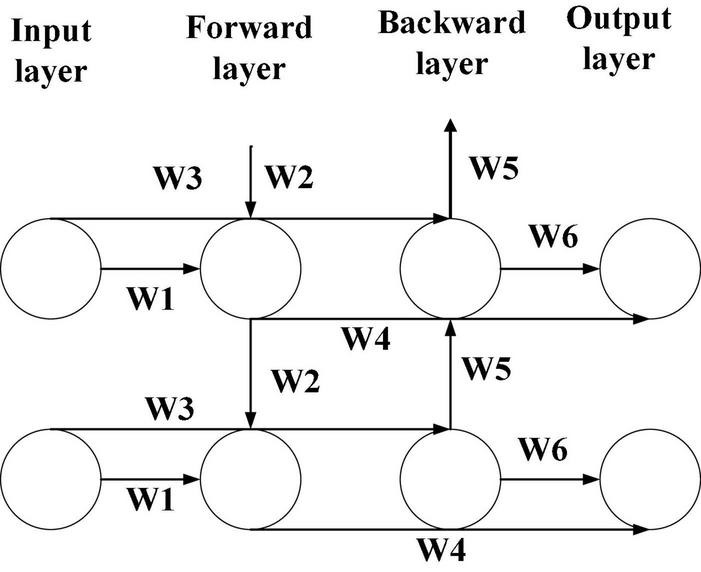
Bidirectional LSTM architecture.

The music sequences in the music library are different. Some music sequences are very long, while some are very short. At present, a bidirectional LSTM network has a good effect in dealing with long-time series problems (Briot, 2021). Bidirectional LSTM is more complex than unidirectional LSTM, which is mainly reflected in the value propagation process. Meanwhile, it needs more training times to optimize the parameters, while the unidirectional LSTM does not need multiple training times. The accuracy of training results of a bidirectional LSTM network is much higher than that of unidirectional LSTM (Jin et al., 2020) after many times of training. Figure 4 displays the LSTM network structure.

The main purpose of the model design is to generate music with a music style. As mentioned earlier, the final music genre style can be distinguished by the strength of music performance. Then, the music of different genres will have different strengths. The range of performance intensity is continuous and large, so it is essential to convert the output value into music strength value and change the output value range through the linear layer.

### Design of Music Style Analysis Model

**FIGURE 5:**
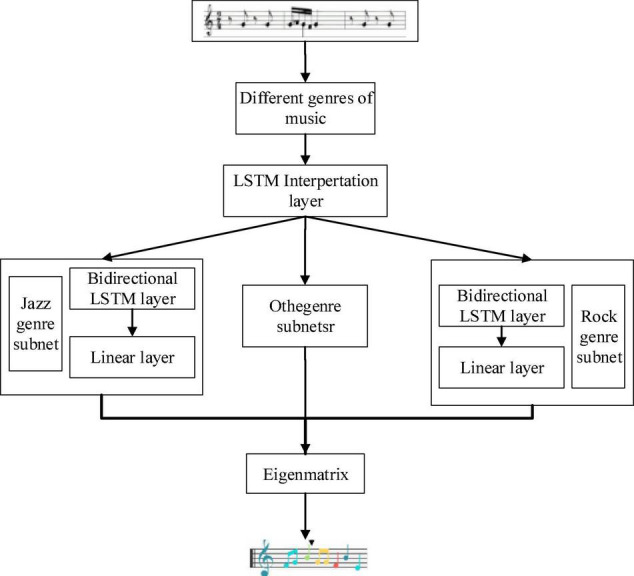
Structure diagram of music style analysis model.

The music style analysis model is mainly adopted to learn more complex style information that cannot be trained in a music genre analysis network. The LSTM neural network is mainly used in this model. Figure 5 is the structural design of the music style analysis model.

The model is to study whether computers can learn and generate different music like people. The model mainly includes the interpretation layer and the subnet of the music genre analysis network. At present, the network is a multi-task learning model. MIDI music can be regarded as a music score. First, it passes through the interpretation layer, and then the output of the interpretation layer is adopted as the input of the music genre analysis network. After it passes through the music genres network, a matrix containing music characteristics will be output. Music analysis networks of different genres will generate matrixes of different genres. The matrix is converted into music that can be played (Shen et al., 2020).

The samples of music data to be analyzed are much fewer than other data, such as user information. The number of categories is relatively large, and there is no obvious distinction rule for each category. Hence, some scholars put forward the Siamese network, which is a similarity measurement method. It maps the input to the target space through a function and compares the similarity in the target space using Euclidean distance (Castillo and Flores, 2021).

In music style analysis, multiple units of the music genre analysis subnet need to be designed in the music style analysis model to better learn different music styles, because music has different genres. Each subnet is connected with the interpretation layer. The output of the interpretation layer is used as the input of the subnet unit. The music style analysis model includes multiple music genre analysis grids, and the biggest difference is an additional interpretation layer. The time of model training is reduced through the interpretation layer. The use of a multitasking mechanism can improve the efficiency of training and deal with the analysis of several kinds of music simultaneously.

A deep belief network combined with the Softmax algorithm is designed to classify the generated music genres. Equation (3) is the standard adopted to evaluate the accuracy of genre classification:


(3) $$Q=\frac{N}{M}\times 100\%$$

*N* represents the number of correctly identified music genres, *M* is the total number of music samples tested, and *Q* represents the accuracy.

The algorithm with the best classification effect among the music genre classification algorithms is selected in the experiment. The algorithm is based on the deep belief network in DL. After the network improvement, Softmax is adopted to predict the genre of music. RBM network belongs to a random network, and its particularity is mainly reflected in two aspects. The first is the probability distribution function. The node state of the network is random. The other is the energy function, which represents the stability of the network state. The greater the energy value is, the stabler the network state is. Equation (4) displays the definition equation of energy in the network.


(4) $$W\,\left(s\right)=-\sum_{i=1}^{n-1}\sum_{j=i+1}^{n}w_{i\,j}\,s_{i}\,s_{j}-\sum_{i=1}^{n}\psi_{i}\,s_{i}$$

ψ_*i*_ represents the value of neurons and *w*_*ij*_ donates the connection weight between neurons.

## Results and Analysis

### Analysis of Experimental Results of Music Style Recognition and Generation

**FIGURE 6:**
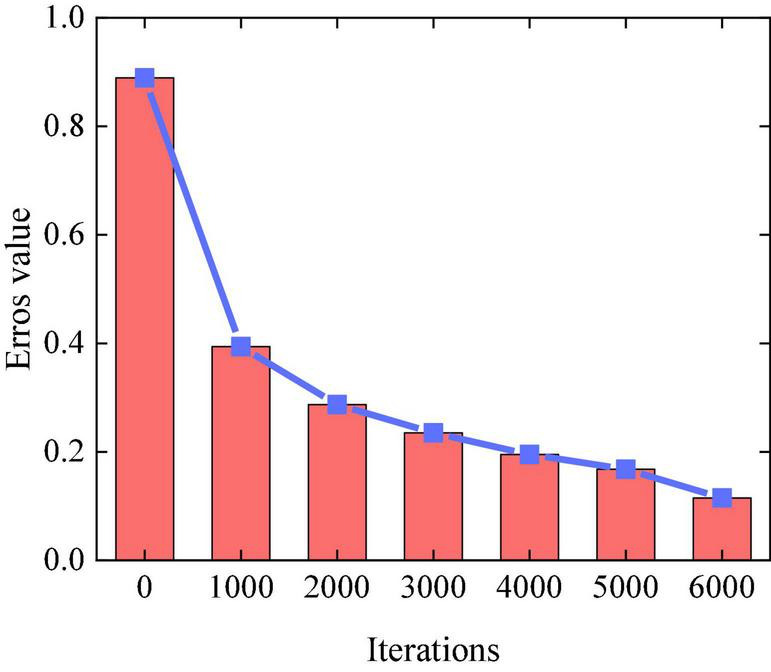
Influence of different iteration times on training effect.

Iterations 1,000, 2,000, 3,000, 4,000, 5,000, and 6,000 are conducted in the experiment to find the best-generated music sequence. The error value of the experiment is increasingly smaller with the increasing iteration times of the LSTM network, which shows that the actual output value is increasingly closer to the target value, and the training results are increasingly accurate. Figure 6 displays the influence of iteration times on the training effect.

**FIGURE 7:**
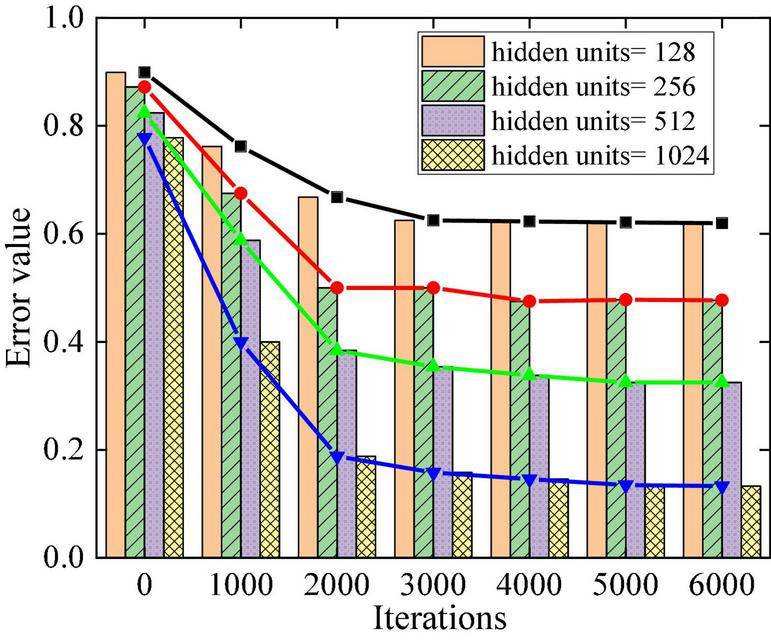
Influence of the number of hidden layer neurons on error.

In the model design, the influence of the number of neurons in the hidden layer on the experiment is analyzed to optimize the model’s parameters. During the experiment, variables are set for the number of hidden layers and the corresponding number of neurons. The number of hidden layers is 1, 2, 3, and 4, and the number of neurons is 1,024, 512, 256, and 128, respectively. Figure 7 displays the effect of hidden layer neurons on experimental error. The number of hidden layers of the network is set to 4 layers, and the number of neurons in each layer is 1,024, 512, 256, and 128, which can minimize the optimal difference of the network training results.

### Analysis of the Generated Music Sequence Spectrum and Original Music Sequence Spectrum

**FIGURE 8:**
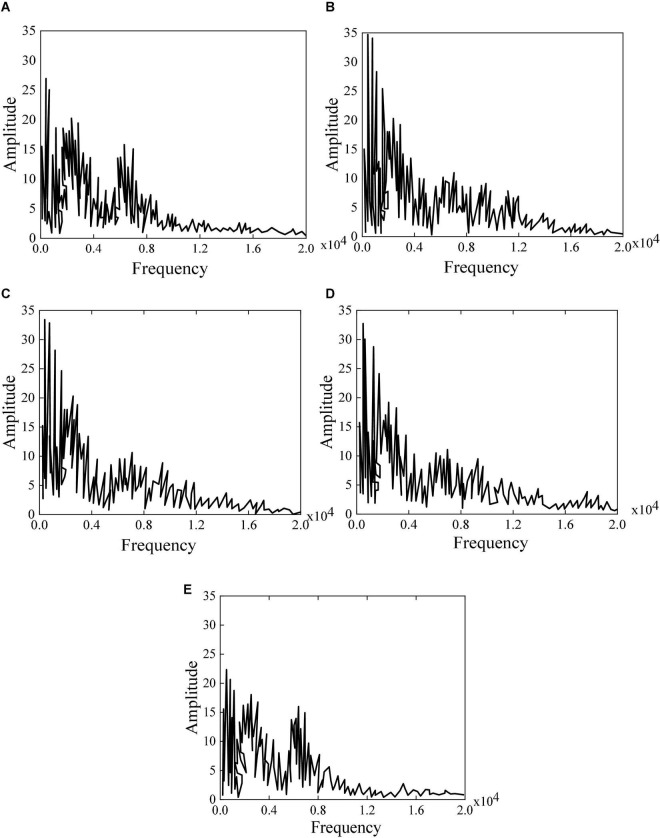
Generated music spectrum and sample spectrum **(A)** original music; **(B)** one hidden layer; **(C)** two hidden layers; **(D)** three hidden layers; **(E)** four hidden layers.

In the experiment, different genres of music are processed by a fast Fourier transform. Then, the obtained music is analyzed by spectrum analysis and sound spectrum analysis to ensure the accuracy of the experimental data. The music sequence spectrum is analyzed during the experiment for the music sequence generated under different hidden layers. Figure 8 displays the generated music spectrum and sample spectrum. It shows that the effect of LSTM on music analysis is still obvious. The music spectrum at the training place is increasingly closer to the original spectrum with the increase of the number of the neural network layer, indicating that the accuracy is increasingly higher. Figure 8A displays the original music. Figure 8B shows that the learned music contains multiple unknown frequencies when there is only one hidden layer. Figure 8C displays that some frequencies do not appear when there are two layers. Figure 8D shows that the generated music sequence file is very similar to the original music sequence file with three layers, but there are still some differences. Figure 8E reveals that the difference between the generated music sequence and the original music sequence is very small when there are four layers, suggesting that the generated music is the most accurate with four hidden layers.

### Generated Music Genre Classification Results

Overall, 5 genres of music are selected, and each music is in MIDI format, with 40 pieces of music of each type.

The classification accuracy of using a deep belief network combined with a Softmax algorithm is as follows (Table 1). The classification accuracy of jazz, classical, rock, country, and disco genres reaches 77.5, 65, 60, 67.5, and 70%, respectively. The analysis of experimental data reveals that the designed algorithm has better classification accuracy than the traditional algorithm. It shows that the music style and genre recognition and generation network in this experiment can generate music of different genres, and the accuracy rate is 60% or more.

**TABLE 1:** Experimental comparative analysis.

Music genre	Design method	Traditional method
Jazz	77.5%	45%
Classical music	65%	34%
Rock	60%	42%
Country	67.5%	55%
Disco	70%	63%

### Analysis of Two Genres of Generative Music

The classification results show that the music style recognition and generation algorithm has a good performance in generating different genres of music. The spectrum of music generated by two different generation models: LSTM and RBM, are analyzed in this experiment.

**FIGURE 9:**
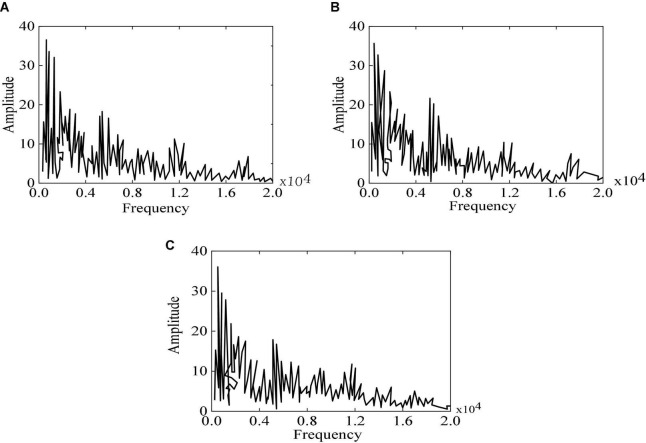
Spectrum diagram of original music, RBM, and the method proposed **(A)** original music; **(B)** RBM; **(C)** the method proposed. **(A)** Is a spectrum diagram of the original music. It reveals that the value of the original sample music does not exceed 20 when the frequency is about 5,000 HZ, but it significantly exceeds in **(B,C)** suggests that the music spectrogram generated by using the method proposed is consistent with the original music spectrogram, both in the overall frequency distribution and the sample frequency distribution.

The experimental results of different algorithms are compared in Figure 9. It reveals that the music generated by traditional RBM is the same as the original music, but the accuracy is not as high as that of the method proposed. Therefore, the designed algorithm can better generate different genres of music.

## Conclusion

In recent years, the rise of DL and machine learning and the rapid progress of computer software and hardware performance have laid a good foundation for the automatic generation of music of different genres. Before that, most researchers used DL networks for music genre classification and recognition but rarely used music generation. Therefore, using the LSTM network for music generation has a certain research significance. The LSTM network has a good effect in dealing with long-time series problems, so it is often used for semantic analysis. On the premise of this foundation, an attempt is made to apply the LSTM network to music generation. There are different genres in music. On the premise of having a certain understanding of the LSTM network, the network model is redesigned using the relevant knowledge. Then, the network can generate multi-task music styles of different genres and improve the training efficiency simultaneously.

Finally, a network that can generate different genre music styles is designed, and the music data in the GTZAN music genre library are used as experimental data for testing. Moreover, the audio and spectrogram of the generated music and the original music are compared and analyzed. The analysis of the spectrum and sound spectrum of the generated music sequence and the original music sequence shows that the network has a good performance in music generation. However, this exploration still has some limitations, and the accuracy of the designed algorithm needs to be further improved. Besides, the music files studied are in MIDI format.

## Data Availability Statement

The raw data supporting the conclusions of this article will be made available by the authors, without undue reservation.

## Ethics Statement

The studies involving human participants were reviewed and approved by the Ethics Committee of Yunnan Minzu University. The patients/participants provided their written informed consent to participate in this study. Written informed consent was obtained from the individual(s) for the publication of any potentially identifiable images or data included in this article.

## Author Contributions

All authors listed have made a substantial, direct, and intellectual contribution to the work, and approved it for publication.

## Conflict of Interest

The authors declare that the research was conducted in the absence of any commercial or financial relationships that could be construed as a potential conflict of interest.

## Publisher’s Note

All claims expressed in this article are solely those of the authors and do not necessarily represent those of their affiliated organizations, or those of the publisher, the editors and the reviewers. Any product that may be evaluated in this article, or claim that may be made by its manufacturer, is not guaranteed or endorsed by the publisher.
